# Cancer Incidence in People With Intellectual Disability and Down Syndrome in Australia: A Cohort Study

**DOI:** 10.1002/cam4.71866

**Published:** 2026-04-29

**Authors:** Julian Trofimovs, Claire M. Vajdic, Preeyaporn Srasuebkul, Sallie‐Anne Pearson, Julian N. Trollor

**Affiliations:** ^1^ National Centre of Excellence in Intellectual Disability Health, Faculty of Medicine and Health University of New South Wales Sydney Australia; ^2^ The Kirby Institute University of New South Wales Sydney Australia; ^3^ Centre for Big Data Research in Health, Faculty of Medicine and Health University of New South Wales Sydney Australia; ^4^ School of Population Health, Faculty of Medicine and Health University of New South Wales Sydney Australia

**Keywords:** cancer, cancer screening, cohort, disability, Down syndrome, intellectual disability, linkage

## Abstract

**Objective:**

There is inconsistent data on cancer risk in people with intellectual disability. Our primary objective was to compare the incidence of cancer in people with and without intellectual disability.

**Methods and Analysis:**

A cohort study using linked population‐based administrative and cancer registry data in New South Wales, Australia 2001–2018. We compared the incidence of cancer in people with intellectual disability and a comparator group without intellectual disability matched for age, sex and residential postcode. We also compared cancer incidence in people with Down syndrome and people with intellectual disability without Down syndrome. We used a flexible parametric survival model accounting for competing risks.

**Results:**

People with intellectual disability had a slightly higher risk of developing any cancer (sub‐hazard ratio, SHR 1.07 [95% CI 1.03 to 1.12]) compared to those without intellectual disability. An increased risk was evident in the 0–14 year (SHR 2.19, 95% CI 1.85 to 2.59) and 15–49 year (SHR 1.34, 95% CI 1.23 to 1.46) age groups, and a decreased risk was observed for those aged 50 years and above (SHR 0.93, 95% CI 0.88 to 0.98). People with intellectual disability had a higher risk of colorectal cancer (SHR 1.32, 95% CI 1.15 to 1.51) and a lower risk of prostate cancer (SHR 0.45, 95% CI 0.38 to 0.53) and melanoma (SHR 0.75, 95% CI 0.64 to 0.88). People with Down syndrome had a significantly higher risk of childhood cancer (SHR 7.94, 95% CI 5.72 to 11.04) compared to those with other intellectual disability, and a similar risk as an adult.

**Conclusions:**

These findings underscore the need for targeted health promotion campaigns and, for adults, customised cancer screening programmes to improve access, acceptability and outcomes for people with intellectual disability.

## 1 Introduction

Intellectual disability is a lifelong impairment of cognition and adaptive behaviour that emerges in childhood [1], affects around 1%–3% of the world population [2, 3], and is associated with increased morbidity and mortality [4, 5, 6]. The underlying causes of intellectual disability are heterogenous, including chromosomal abnormality, gene mutation, environmental factors and prenatal factors [7]. The exact cause, however, is not identifiable for most people with intellectual disability [8].

Cancer is a leading cause of morbidity and mortality worldwide. In Australia alone, approximately 169,000 new cancer cases are projected in 2024, rising to 209,000 cases annually by 2034. Five‐year survival rates have improved substantially over time, from 55% in 1991–1995 to 71% in 2019–2020 [9]. Parallel improvements in healthcare have led to increased life expectancy for people with intellectual disability [10], resulting in greater rates of diagnosis with age‐related conditions such as cancer. Although research into cancer incidence in people with intellectual disability has expanded in recent decades, population‐based studies assessing the risk of any cancer in this population have presented contradictory findings (Table S1). Some studies report similar overall cancer incidence rates to those of the general population [11, 12], while others report elevated risks for specific cancer types. Epidemiological evidence demonstrates consistently elevated cancer risks among people with intellectual disability across multiple organ systems, including cancers of the gallbladder, thyroid gland, oesophagus, stomach, small intestine, colon, pancreas, uterus, kidney and central nervous system, with additional reports of increased incidence, though less consistent, for testicular, ovarian, skin, brain and ocular malignancies. Unique biological mechanisms may contribute to cancer risk among people with intellectual disability, including chromosomal abnormalities and genetic mutations associated with syndromic intellectual disability [13, 14]. The association between Down syndrome and elevated risk of leukaemia is well established and attributed to constitutional trisomy 21–associated gene dosage effects on haematopoiesis, particularly involving genes such as *GATA1* and *RUNX1* [15, 16].

We aimed to estimate the incidence of cancer in people with intellectual disability using linked administrative health data from New South Wales, Australia. We compared these rates to a matched population‐based comparator group without intellectual disability and explored differences in cancer incidence between people with Down syndrome and other forms of intellectual disability.

## 2 Methods

### 2.1 Study Design, Data Sources and Study Population

We conducted a cohort study using longitudinal administrative health data from New South Wales (NSW), Australia [17]. Anonymised individual‐level data were sourced from a linked population‐based data asset containing health and service records of people with intellectual disability and matched comparators without intellectual disability. The NSW Centre for Health Record Linkage (CheReL) identified people with intellectual disability using intellectual disability flags from disability service datasets and ICD‐9 and ICD‐10 codes from health records (Table S2). This multi‐source approach is consistent with previous population‐based studies using administrative data to identify intellectual disability [18, 19, 20, 21, 22]. CheReL and the Australian Institute for Health and Welfare (AIHW) performed probabilistic data linkage.

**FIGURE 1:**
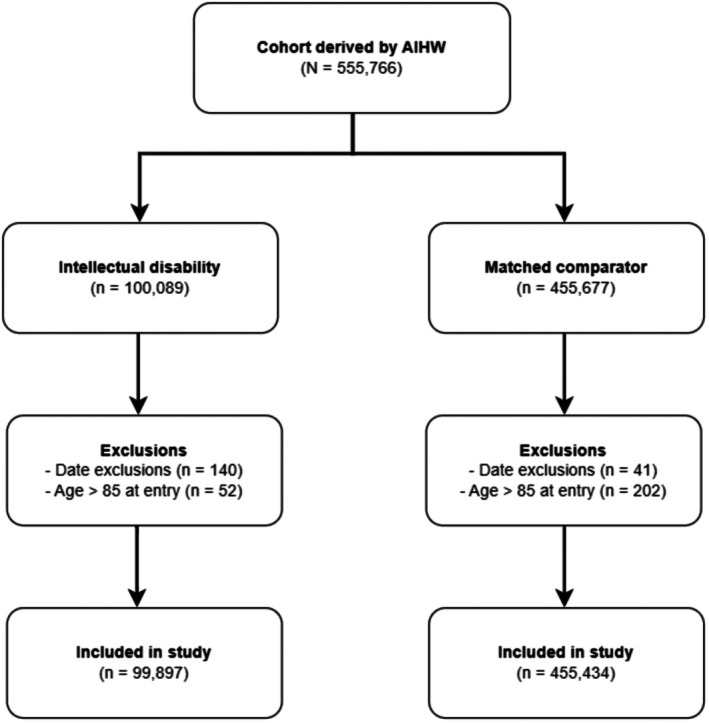
Study flow chart.

The intellectual disability cohort comprised people alive and younger than 85 years as of July 1, 2001. We identified diagnoses of intellectual disability from one or more of the following NSW datasets: disability services, specialist public school support services, corrective services, Ombudsman and Public Guardian services, ambulatory mental health services, hospital inpatient services and emergency department presentations between 1 January 2001 and 31 December 2018. As intellectual disability is a lifelong condition, individuals were considered to have intellectual disability from birth, regardless of when identification occurred in the administrative records. Disability services records require formal assessment and documentation of intellectual disability to access services, while hospital coding is likely to have high specificity given associated stigma [23, 24]. We excluded people who had died or turned 85 before 1 July 2001, and those born after 31 December 2017 ensuring a minimum potential follow‐up of 1 year for all participants (Figure 1). Within the intellectual disability cohort, we identified a subpopulation of people with Down syndrome using diagnostic codes from the linked datasets (ICD9 code 758.0, ICD10 Q90 or ICD10AM code U88.2).

Comparators were people without intellectual disability randomly selected from all Australians eligible for Medicare, a national, publicly funded, universal insurance scheme for health care services and medicines. Comparators were frequency‐matched to people with intellectual disability by age, sex and residential postcode at a ratio of up to 5:1.

### 2.2 Outcome Ascertainment

We identified invasive cancer diagnoses from linked NSW Cancer Registry records. We classified any cancer, all haematological cancers, all solid cancers, screen‐detectable cancers (colorectal, breast, cervical), as well as common cancers (melanoma, lung and liver; Table S3). Cancer is a notifiable disease in Australia, and the NSW Cancer Registry is considered high‐quality. We obtained dates of death from the Registry of Births, Deaths and Marriages. Some invasive cancer cases could not be classified as either solid or haematological malignancies, resulting in totals that may not sum across categories.

### 2.3 Covariate Assessment

Sex, a study design variable, was a covariate. We created two directed acyclic graphs (Figures S1 and S2), which guided our selection of covariates. We used residential postcode to derive two area‐level covariates: an area‐level socio‐economic indicator (Index of Relative Socioeconomic Disadvantage, IRSD) [25] and a remoteness indicator (Accessibility/Remoteness Index of Australia, ARIA^+^) [26]. We classified Aboriginal or Torres Strait Islander status as ever/never, based on self‐reported status in several of the administrative datasets and included this variable as a covariate.

### 2.4 Statistical Analysis

We used flexible parametric survival models to evaluate the association between intellectual disability and cancer incidence and generated sub‐hazard ratios (SHRs) with 95% confidence intervals (CIs) [27]. Follow‐up for cancer began on 1 July 2001, or the date of birth, whichever was later, and ended on the date of first cancer diagnosis, death, 85th birthday or 31 December 2018, whichever came first. If the date of death and the date of cancer diagnosis were identical, the cancer diagnosis was counted. Given the excess mortality in people with intellectual disability [6, 28], we employed a competing risks model with death as a competing event and used age (0–85 years) as the timescale.

We stratified analyses into three age groups (0–14, 15–50 and 50+ years), based on the Australian definition of childhood cancers and survival curves for adults. Individuals contributed follow‐up time to each age group as they aged through the study period. In a secondary, exploratory analysis, we compared cancer incidence in people with Down syndrome and those with other forms of intellectual disability.

A complete case analysis was performed, excluding participants with missing data on area‐level variables (remoteness, socioeconomic disadvantage). Intellectual disability status and cancer outcomes were available for all participants. This resulted in the exclusion of 52,231 people, yielding a final analytical sample of 503,010.

We performed all analyses using Stata version 18.0 (StataCorp, College Station, TX, USA).

This report aligns with the Reporting of Studies Conducted using Observational Routinely‐collected Health Data (RECORD) Statement.

### 2.5 Study Conduct

Access to data provided by the data custodians is subject to approval. All inferences, opinions and conclusions drawn from this research are those of the authors and do not reflect the opinions or policies of the data custodians.

### 2.6 Patient and Public Involvement

Patients or the public were not involved in the design, conduct or reporting of our research. We will prepare easy‐read summaries of the findings for dissemination to people with intellectual disability.

### 2.7 Use of Generative AI

Generative AI tools were used by only two authors to improve readability and clarity. Those tools were not used by the remaining authors and were not used to generate, analyse, interpret or create any scientific content of the manuscript.

## 3 Results

### 3.1 Description of the Cohort

Our cohort consisted of 555,331 people, including 99,897 (18%) with intellectual disability (4492 with Down syndrome) and 455,434 (82%) without intellectual disability (Table 1). The median age at the start of follow‐up was 2 (IQR 0–19) for people with intellectual disability, 10 (IQR 0–34) for people with Down syndrome, 2 (IQR 0–18) for people with other intellectual disability, and 3 (IQR 0–20) for people without intellectual disability. Compared to those with intellectual disability other than Down syndrome, people with Down syndrome were proportionally older at the start of follow‐up: 11.9% versus 6.9% aged 45–65 years. Comparison of baseline characteristics for included and excluded individuals showed no meaningful differences for key variables (data not shown).

**TABLE 1:** Cohort characteristics at baseline for people with and without intellectual disability.

	Intellectual disability	Intellectual disability other than Down syndrome	Down syndrome	Matched comparator without intellectual disability
	99,897 (18%)	95,405 (17.2%)	4492 (0.81%)	455,434 (82.0%)
Age at study start, years				
0–14	70,825 (70.9%)	68,356 (71.6%)	2469 (55.0%)	317,391 (69.7%)
15–18	3450 (3.5%)	3283 (3.4%)	167 (3.7%)	17,812 (3.9%)
19–24	4170 (4.2%)	3891 (4.1%)	279 (6.2%)	20,845 (4.6%)
25–44	12,903 (12.9%)	11,905 (12.5%)	998 (22.2%)	58,189 (12.8%)
45–64	7087 (7.1%)	6553 (6.9%)	534 (11.9%)	33,884 (7.4%)
65+	1462 (1.5%)	1417 (1.5%)	45 (1.0%)	7313 (1.6%)
Sex				
Female	33,730 (33.8%)	31,626 (33.1%)	2104 (46.8%)	159,129 (34.9%)
Male	66,167 (66.2%)	63,779 (66.9%)	2388 (53.2%)	296,305 (65.1%)
Residential remoteness				
Major cities	57,721 (57.8%)	55,111 (57.8%)	2610 (58.1%)	260,784 (57.3%)
Inner regional	22,814 (22.8%)	21,889 (22.9%)	925 (20.6%)	111,038 (24.4%)
Outer regional	7339 (7.3%)	7038 (7.4%)	301 (6.7%)	38,686 (8.5%)
Remote & very remote	802 (0.8%)	780 (0.8%)	22 (0.5%)	3838 (0.8%)
Missing	11,221 (11.2%)	10,587 (11.1%)	634 (14.1%)	41,088 (9.0%)
Area‐level Index of Relative Socioeconomic Disadvantage (IRSD)				
First quintile (most disadvantaged)	24,629 (24.7%)	23,749 (24.9%)	880 (19.6%)	102,743 (22.6%)
Second quintile	22,057 (22.1%)	21,212 (22.2%)	845 (18.8%)	99,407 (21.8%)
Third quintile	21,139 (21.2%)	20,256 (21.2%)	883 (19.7%)	97,831 (21.5%)
Fourth quintile	15,358 (15.4%)	14,629 (15.3%)	729 (16.2%)	78,585 (17.3%)
Fifth quintile (least disadvantaged)	11,645 (11.7%)	10,851 (11.4%)	794 (17.7%)	62,115 (13.6%)
Missing	5069 (5.1%)	4708 (4.9%)	361 (8.0%)	14,753 (3.2%)

*Note:* Missing data are presented for descriptive purposes. Subsequent analyses were restricted to participants with complete data on all variables.

Within the intellectual disability cohort, those with Down syndrome were more likely to be female, less likely to reside in areas of highest socioeconomic disadvantage.

### 3.2 Cancer Incidence

A total of 14,136 people were diagnosed with one or more notifiable cancers during follow‐up, including 2474 (18%) people with intellectual disability. We observed 11,863 deaths, 5458 in people with intellectual disability and 6405 among matched comparators. The median follow‐up time was 16.9 years for people with intellectual disability and 17.0 years for matched comparators. The age‐adjusted cancer incidence rates were 1.78 and 1.79 cases per 1000 person‐years in people with intellectual disability and matched comparators, respectively.

Among those diagnosed with cancer, people with intellectual disability were diagnosed at a younger age compared to those without intellectual disability (Table S4). The greatest difference was observed for haematological cancers, where people with intellectual disability were more likely to be diagnosed during childhood (ages 0–14 years): 17.8% compared to 7.1% of matched comparators. Compared to people without intellectual disability, people with intellectual disability had a higher risk of any cancer at any age, during childhood and as adults aged 15–49 years (Table 2). For adults aged 50+ years, the opposite was observed, and people with intellectual disability had a lower risk of any cancer.

**TABLE 2:** Hazard ratios for any cancer, solid cancer and haematological cancer by age group.

Cancer group and attained age	Number of incident cases	SHR (95% CI)
Any age		
Any cancer	13,024	1.07 (1.03, 1.12)
Solid cancer	11,134	1.02 (0.97, 1.07)
Haematological cancer	1327	1.18 (1.03, 1.36)
0–14 years		
Any cancer	680	2.19 (1.85, 2.59)
Solid cancer	329	2.50 (1.98, 3.16)
Haematological cancer	247	2.00 (1.51, 2.66)
15–49 years		
Any cancer	3031	1.34 (1.23, 1.46)
Solid cancer	2562	1.26 (1.14, 1.28)
Haematological cancer	361	1.34 (1.05, 1.70)
50+ years		
Any cancer	9313	0.93 (0.88, 0.98)
Solid cancer	8243	0.90 (0.84, 0.95)
Haematological cancer	719	0.89 (0.72, 1.10)

*Note:* All models were adjusted for sex, Indigenous status, remoteness and area‐level socio‐economic status. Age is a timescale variable for all analyses.

Abbreviations: CI, confidence interval; SHR, sub hazard ratio.

Considering solid cancers, compared to people without intellectual disability, people with intellectual disability were at increased risk during childhood and as adults aged 15–49 years, and they were at decreased risk as adults aged 50+ years (Table 2). When all ages were combined, there was no difference in solid cancer risk for people with intellectual disability. People with intellectual disability were at a higher risk of developing haematological cancer in all age categories except for adults aged 50+ (Table 2).

Considering specific cancer types, people with intellectual disability had a higher risk of colorectal cancer and a lower risk of prostate cancer and melanoma (Table 3).

**TABLE 3:** Hazard ratios for specific cancer types.

Cancer type	Number of incident cases	SHR (95% CI)
Prostate	1919	0.45 (0.38–0.53)
Breast	1633	1.03 (0.90–1.18)
Melanoma	1394	0.75 (0.64–0.88)
Colorectal	1329	1.32 (1.15–1.51)
Lung	935	0.95 (0.79–1.13)
Liver	162	0.92 (0.60–1.42)
Cervical	88	0.92 (0.51–1.65)

*Note:* All models were adjusted for sex (except for prostate, breast and cervical cancers), Indigenous status, remoteness and area‐level socio‐economic status. Age is a timescale variable for all analyses.

Abbreviations: CI, confidence interval; SHR, sub hazard ratio.

### 3.3 Secondary Analysis: People With Down Syndrome

Of the 4492 people with Down syndrome, 145 were diagnosed with cancer, and 751 died during follow‐up. For people with other intellectual disability, 2329 were diagnosed with cancer and 4707 died. The median follow‐up time was 16.9 years for both groups. The age‐adjusted cancer incidence rates were 2.52 and 1.75 cases per 1000 person‐years in people with Down syndrome and those with other intellectual disability, respectively. Twenty‐four percent of people with Down syndrome and cancer were diagnosed at ages 0–4, compared to 4.7% of those with other intellectual disability (Table S5).

People with Down syndrome had a significantly higher risk of childhood cancer (SHR 7.94, 95% CI 5.72 to 11.04) compared to those with other intellectual disabilities (Table S6). At other ages, the risk of cancer was similar between the two groups.

## 4 Discussion

We found a higher incidence of cancer in people with intellectual disability compared to people without intellectual disability during childhood and adulthood up to 49 years of age, for both solid and haematological cancers. In contrast, for people with intellectual disability aged 50 years and above, the incidence of cancer was lower. Notably, people with intellectual disability had an increased risk of colorectal cancer and decreased risks of both prostate cancer and melanoma.

These findings contrast with previous population‐based studies that reported a lower or similar incidence of cancer for people with intellectual disability compared to the general population [11, 29, 30]. Several factors may explain this discordance. Firstly, many population‐based studies include a large proportion of people with Down syndrome who have a decreased rate of solid tumours [29, 31]. Secondly, earlier studies often relied on institutionalised populations or outdated administrative datasets that may not reflect the broader contemporary population of people with intellectual disability, particularly those living in the community [11, 29]. Thirdly, methodological differences—such as shorter follow‐up times, less comprehensive data linkage (e.g., not including cancer registries) and differences in matching criteria or adjustment for confounders—may have limited the accuracy of earlier incidence estimates. Most previous studies calculated standardised incidence ratios (SIRs), which do not account for competing risks or allow adjustment for important confounders. For example, Patja et al. [29] lacked data on socioeconomic status, and Sullivan et al. [11] did not stratify cancer risk by age. In contrast, our use of sub‐hazard ratios from competing risks models accounted for the excess mortality in this population and allowed greater confounder adjustment, potentially explaining the higher cancer incidence observed in our study.

Similar to previous studies [11, 29, 32], we found lower rates of prostate cancer and invasive melanoma among people with intellectual disability compared to the general population. Several factors could explain these findings. For prostate cancer, rather than a biological explanation, the lower incidence may suggest that screening tests, including the prostate‐specific antigen (PSA) test, are recommended less commonly for people with intellectual disability compared to those without. Additionally, as urinary symptoms are commonly experienced by people with intellectual disability, their potential association with prostate cancer may be overlooked and less assertively investigated [33]. For melanoma, behavioural differences may explain the reduced risk for people with intellectual disability. Intense, intermittent sun exposure, particularly in childhood and adolescence, is the strongest risk factor for cutaneous melanoma [34]. The mechanisms underlying these associations require further investigation.

The higher cancer incidence in childhood (ages 0–14) among people with intellectual disability may be partly explained by the presence of certain genetic syndromes, such as Down syndrome, which are associated with markedly increased risks of specific childhood cancers, particularly leukaemia [11]. Beyond genetic predisposition, early‐life exposures and perinatal complications commonly associated with intellectual disability—such as in utero exposure to environmental toxins, low birth weight, preterm birth and disrupted immune development—have been implicated in childhood cancer susceptibility, particularly leukaemia [35, 36].

Among adolescents and young adults, the elevated incidence of cancer in people with intellectual disability may reflect both genetic predisposition and disparities in health monitoring or access to timely care [37]. The transition from paediatric to adult healthcare services is a known point of vulnerability for people with intellectual disability, often marked by gaps in service coordination and decreased access to preventive care [38]. These recognised barriers to care can limit engagement with early intervention pathways and may contribute to a higher cancer burden in this age group. Furthermore, people with intellectual disability are more likely to experience early onset of chronic health conditions such as severe mental illness, obesity and diabetes, which are associated with increased cancer risk and may shift the age of onset earlier than in the general population [4, 39, 40]. Obesity and diabetes are especially relevant for people with intellectual disability, given their elevated exposure to risk factors, including poor diet quality, physical inactivity and psychotropic medications use [41, 42, 43]. These health conditions may contribute to elevated cancer risk, although we could not examine their role in the absence of primary care data. Coordinated preventive care frameworks that prioritise dietary quality, active living and the safe monitoring of medication side effects warrant further investigation [44, 45].

Our findings highlight the need for a nuanced and age‐sensitive approach to cancer prevention‐related care for people with intellectual disability. In childhood, increased clinical awareness is needed to detect malignancies early, especially in those with known genetic syndromes, to maximise treatment choice and prognosis. For adolescents and adults, integrated strategies must prioritise tailored health promotion that addresses modifiable lifestyle factors—such as smoking, diet, physical activity and the metabolic side effects of medications—alongside age‐appropriate cancer screening programmes. Strengthening transitions between paediatric and adult care, improving access to preventive services and equipping support settings to actively promote healthy lifestyles are essential steps toward reducing cancer disparities in this population.

## 5 Strengths and Limitations

Our study has several strengths, including comprehensive coverage of people with intellectual disability, achieved through integration of multiple record sources and robust data linkage. We also used population registries to identify cancers and deaths. The use of advanced statistical models, such as flexible parametric survival models and competing risk models, enhances the robustness of our findings. The competing risk model, accounting for death as a competing event, provides a more accurate estimation of cancer incidence by appropriately handling the possibility that individuals with intellectual disability may die before developing cancer, thereby reducing bias in the cumulative incidence estimates.

As the research is based on data from New South Wales, Australia, the generalisability of our findings to other regions or countries with different population demographics and healthcare systems, including cancer screening practices, may be limited. We were unable to stratify by severity of intellectual disability as this information was not routinely captured; it is possible that disability severity could affect both cancer risk, access to and receipt of preventative health care, noting the need to prioritise life‐threatening and acute conditions. Despite using health, social and justice records to identify intellectual disability, individuals with mild intellectual disability are likely to be under‐represented. Therefore, individuals with mild or borderline intellectual disability may be included in the comparator group, potentially underestimating the risk of cancer in people with intellectual disability. Although the CHeReL reports high linkage quality [46], probabilistic data linkage results in false matches and missed links, which would likely result in bias toward the null. Approximately 9% of individuals were excluded due to missing area‐level data, requiring a complete case analysis and potentially introducing a systematic bias if the missingness was not at random. Comparison of complete and incomplete cases showed that excluded individuals with intellectual disability were younger than those included, while excluded comparators were younger, more likely to be female and less likely to live in the most disadvantaged areas (Table S7). Multiple imputation was considered; however, the missing area‐level variables (remoteness, socioeconomic disadvantage) would require residential address data for imputation that were not available in our dataset. Although we examined the most populous Australian state for nearly two decades, the number of incident cancers was too small to examine rarer cancers that have been observed to occur at higher incidence in people with intellectual disability. Additionally, our analysis could not account for individual lifestyle behaviours, environmental exposures or genetic predispositions, as these data are not collected at the population level.

Lower cancer screening rates and diagnostic practices for people with intellectual disability could lead to detection bias and result in a reduced incidence of cancer and older age at diagnosis [30]. Our censor was 2001 or the date of birth (whichever was later), and this likely resulted in the older age at the start of follow‐up for those with a genetic disorder (e.g., Down syndrome), who were more likely to be represented in the historical disability service and hospital records than people with milder forms of disability. Due to the administrative data sources used for disability identification (primarily schools and disability services), person‐time is concentrated in childhood and early adulthood. Thus, estimates for older age groups are based on fewer person‐years and should be interpreted with caution. Despite this, our models detected a significantly higher SHR for childhood cancers in this group compared to others with intellectual disability, although our estimates are likely conservative. Notably, incidence rates became comparable as the cohorts progressed into adulthood.

Differences in cancer risk and healthcare access between socioeconomic groups were likely inadequately captured by the area‐based measure of socioeconomic disadvantage. Furthermore, we had no data on ethnicity. Even with the use of advanced statistical models, our findings should be interpreted cautiously, considering the possibility of unadjusted confounding.

## 6 Conclusion

Our findings highlight a markedly elevated risk of cancer in children with intellectual disability, particularly those with Down syndrome, underscoring the importance of clinical awareness, timely diagnosis and tailored surveillance as well as primary prevention. Among adults with intellectual disability, the increased incidence of certain cancers, such as colorectal cancer, points to the need for targeted health promotion and equitable access to cancer screening programmes. Future research should examine how the severity and type of intellectual disability influence cancer risk and outcomes across the lifespan.

## Author Contributions

**Julian Trofimovs:** conceptualization, formal analysis, investigation, project administration, visualization, writing – original draft, writing – review and editing. **Claire M. Vajdic:** conceptualization, funding acquisition, investigation, methodology, supervision, validation, writing – review and editing. **Preeyaporn Srasuebkul:** conceptualization, writing – original draft, methodology, writing – review and editing, formal analysis, supervision, data curation, software, project administration, investigation, resources, visualization, validation. **Sallie‐Anne Pearson:** conceptualization, funding acquisition, investigation, writing – review and editing. **Julian N. Trollor:** conceptualization, investigation, funding acquisition, resources, writing – review and editing.

## Funding

The Australian National Health and Medical Research Council funded this work (GNT 1123033 and GNT2009771). The Aspen Medical Foundation also provided financial support.

## Disclosure

Patient and public involvement: People with intellectual disability were not involved in the design, reporting, but will be involved in dissemination plans of this research. Refer to the Methods section for further details.

## Ethics Statement

The NSW Population & Health Services Research Ethics Committee (HREC/17/CIPHS/49), the Australian Institute of Health and Welfare Ethics Committee (AIHW; EO2017/5/404), the ACT Health Ethics Committee (ETH.11.17.262), the Calvary Public Hospital Bruce Ethics Committee (53–2017), the Corrective Services NSW Ethics Committee (approval date 19/07/2018) and the NSW Department of Education Ethics Committee (SERAP2017600) approved the study.

## Conflicts of Interest

P.S. is a consultant for the Australian Institute of Health and Welfare (AIHW) but the views expressed in this manuscript are those of the authors and do not necessarily reflect the views or policies of AIHW. The other authors declare no conflicts of interest.

## Supporting information


**Table S1:** Summary of Population‐Based Studies on Cancer Incidence in People with Intellectual Disabilities.
**Table S2:** Diagnostic codes for intellectual disability.
**Table S3:** Cancer groupings.
**Table S4:** Characteristics of people with cancer, by cancer type and intellectual disability status.
**Table S5:** Cohort characteristics of people with intellectual disability with cancer by Down syndrome status.
**Table S6:** Hazard ratios of any cancer in people with Down syndrome.
**Table S7:** Cohort characteristics of individuals with and without missing information.
**Figure S1:** Directed acyclic graph showing proposed causal relationships between predictor variables and the outcome diagnosis for the intellectual disability cohort. Figure made using DAGitty (http://www.dagitty.net/).
**Figure S2:** Directed acyclic graph showing proposed causal relationships between predictor variables and the outcome diagnosis for the Down syndrome cohort. Figure made using DAGitty (http://www.dagitty.net/).

## Data Availability

The data that support the findings of this study are available on request from the corresponding author. The data are not publicly available due to privacy or ethical restrictions.

## References

[1] P. K. Maulik , M. N. Mascarenhas , C. D. Mathers , T. Dua , and S. Saxena , “Prevalence of Intellectual Disability: A Meta‐Analysis of Population‐Based Studies,” Research in Developmental Disabilities 32, no. 2 (2011): 419–436. 21236634 10.1016/j.ridd.2010.12.018

[2] J. Bourke , R. Sanders , J. Jones , M. Ranjan , K. Wong , and H. Leonard , “Intellectual Disability and Autism Prevalence in Western Australia: Impact of the NDIS,” Frontiers in Psychiatry 15 (2024): 1359505. 38832329 10.3389/fpsyt.2024.1359505 PMC11145759

[3] S.‐A. Cooper , “Definitions, Classification, and Epidemiology of Intellectual Disability,” in Oxford Textbook of the Psychiatry of Intellectual Disability, ed. S. Bhaumik and R. Alexander (Oxford University Press, 2020).

[4] F. J. Hosking , I. M. Carey , S. M. Shah , et al., “Mortality Among Adults With Intellectual Disability in England: Comparisons With the General Population,” American Journal of Public Health 106, no. 8 (2016): 1483–1490. 27310347 10.2105/AJPH.2016.303240 PMC4940652

[5] P. Liao , C. Vajdic , J. Trollor , and S. Reppermund , “Prevalence and Incidence of Physical Health Conditions in People With Intellectual Disability—A Systematic Review,” PLoS One 16, no. 8 (2021): e0256294. 34428249 10.1371/journal.pone.0256294 PMC8384165

[6] J. Trollor , P. Srasuebkul , H. Xu , and S. Howlett , “Cause of Death and Potentially Avoidable Deaths in Australian Adults With Intellectual Disability Using Retrospective Linked Data,” BMJ Open 7, no. 2 (2017): e013489. 10.1136/bmjopen-2016-013489 PMC5306525 28179413

[7] J. C. Harris , Intellectual Disability: Understanding Its Development, Causes, Classification, Evaluation, and Treatment (Oxford University Press, 2005).

[8] A. Rauch , J. Hoyer , S. Guth , et al., “Diagnostic Yield of Various Genetic Approaches in Patients With Unexplained Developmental Delay or Mental Retardation,” American Journal of Medical Genetics. Part A 140, no. 19 (2006): 2063–2074. 16917849 10.1002/ajmg.a.31416

[9] Australian Institute of Health and Welfare (AIHW) , “Cancer Data in Australia,” (2024), https://www.aihw.gov.au/reports/cancer/cancer‐data‐in‐australia/contents/overview.

[10] A. M. Coppus , “People With Intellectual Disability: What Do We Know About Adulthood and Life Expectancy?,” Developmental Disabilities Research Reviews 18, no. 1 (2013): 6–16. 23949824 10.1002/ddrr.1123

[11] S. G. Sullivan , R. Hussain , T. Threlfall , and A. H. Bittles , “The Incidence of Cancer in People With Intellectual Disabilities,” Cancer Causes & Control 15, no. 10 (2004): 1021–1025. 15801486 10.1007/s10552-004-1256-0

[12] L. M. Ward , S. A. Cooper , F. Sosenko , et al., “Population‐Based Cancer Incidence and Mortality Rates and Ratios Among Adults With Intellectual Disabilities in Scotland: A Retrospective Cohort Study With Record Linkage,” BMJ Open 14, no. 8 (2024): e084421. 10.1136/bmjopen-2024-084421 PMC11331995 39142671

[13] A. Asim , A. Kumar , S. Muthuswamy , S. Jain , and S. Agarwal , “Down Syndrome: An Insight of the Disease,” Journal of Biomedical Science 22, no. 1 (2015): 41. 26062604 10.1186/s12929-015-0138-y PMC4464633

[14] J. Wechsler , M. Greene , M. A. McDevitt , et al., “Acquired Mutations in GATA1 in the Megakaryoblastic Leukemia of Down Syndrome,” Nature Genetics 32, no. 1 (2002): 148–152. 12172547 10.1038/ng955

[15] M. Labuhn , K. Perkins , S. Matzk , et al., “Mechanisms of Progression of Myeloid Preleukemia to Transformed Myeloid Leukemia in Children With Down Syndrome,” Cancer Cell 36, no. 3 (2019): 340. 31526763 10.1016/j.ccell.2019.08.014

[16] A. Roy , G. Cowan , A. J. Mead , et al., “Perturbation of Fetal Liver Hematopoietic Stem and Progenitor Cell Development by Trisomy 21,” National Academy of Sciences of the United States of America 109, no. 43 (2012): 17579–17584. 10.1073/pnas.1211405109 PMC3491522 23045701

[17] S. Reppermund , P. Srasuebkul , C. M. Vajdic , S. A. Pearson , R. E. Moorin , and J. N. Trollor , “Cohort Profile: Understanding Health Service System Needs for People With Intellectual Disability Using Linked Data in New South Wales, Australia,” Epidemiology and Health 46 (2024): e2024054. 38901827 10.4178/epih.e2024054 PMC11573485

[18] S. Azimi , F. Lima , L. Slack‐Smith , et al., “Factors Associated With Dental Hospitalisations in Children With Intellectual Disability or Autism Spectrum Disorder: A Western Australian Population‐Based Retrospective Cohort Study,” Disability and Rehabilitation 44, no. 19 (2022): 5495–5503. 34148478 10.1080/09638288.2021.1936662

[19] K. Brameld , K. Spilsbury , L. Rosenwax , H. Leonard , and J. Semmens , “Use of Health Services in the Last Year of Life and Cause of Death in People With Intellectual Disability: A Retrospective Matched Cohort Study,” BMJ Open 8, no. 2 (2018): e020268. 10.1136/bmjopen-2017-020268 PMC5855242 29478966

[20] M. Cuypers , H. Tobi , J. Naaldenberg , and G. L. Leusink , “Linking National Public Services Data to Estimate the Prevalence of Intellectual Disabilities in The Netherlands: Results From an Explorative Population‐Based Study,” Public Health 195 (2021): 83–88. 34062276 10.1016/j.puhe.2021.04.002

[21] E. Lin , R. Balogh , B. Isaacs , et al., “Strengths and Limitations of Health and Disability Support Administrative Databases for Population‐Based Health Research in Intellectual and Developmental Disabilities,” Journal of Policy and Practice in Intellectual Disabilities 11, no. 4 (2014): 235–244.

[22] S. Reppermund , P. Srasuebkul , T. Heintze , et al., “Cohort Profile: A Data Linkage Cohort to Examine Health Service Profiles of People With Intellectual Disability in New South Wales, Australia,” BMJ Open 7, no. 4 (2017): e015627. 10.1136/bmjopen-2016-015627 PMC5541414 28404614

[23] A. Ali , M. King , A. Strydom , and A. Hassiotis , “Self‐Reported Stigma and Its Association With Socio‐Demographic Factors and Physical Disability in People With Intellectual Disabilities: Results From a Cross‐Sectional Study in England,” Social Psychiatry and Psychiatric Epidemiology 51, no. 3 (2016): 465–474. 26498927 10.1007/s00127-015-1133-z

[24] J. Bourke , K. Wong , and H. Leonard , “Validation of Intellectual Disability Coding Through Hospital Morbidity Records Using an Intellectual Disability Population‐Based Database in Western Australia,” BMJ Open 8, no. 1 (2018): e019113. 10.1136/bmjopen-2017-019113 PMC5786126 29362262

[25] Australian Bureau of Statistics (ABS) , “Socio‐Economic Indexes for Areas (SEIFA), Australia Methodology,” (2021), https://www.abs.gov.au/methodologies/socio‐economic‐indexes‐areas‐seifa‐australia‐methodology/2021#introduction.

[26] Australian Bureau of Statistics (ABS) , Australian Statistical Geography Standard (ASGS), 3rd ed. (Australian Bureau of Statistics (ABS), 2021), https://www.abs.gov.au/statistics/standards/australian‐statistical‐geography‐standard‐asgs‐edition‐3/jul2021‐jun2026#asgs‐diagram.

[27] P. Royston and P. C. Lambert , Flexible Parametric Survival Analysis Using Stata: Beyond the Cox Model (Stata Press, 2011).

[28] P. Heslop , P. S. Blair , P. Fleming , M. Hoghton , A. Marriott , and L. Russ , “The Confidential Inquiry Into Premature Deaths of People With Intellectual Disabilities in the UK: A Population‐Based Study,” Lancet 383, no. 9920 (2014): 889–895. 24332307 10.1016/S0140-6736(13)62026-7

[29] K. Patja , P. Eero , and M. Iivanainen , “Cancer Incidence Among People With Intellectual Disability,” Journal of Intellectual Disability Research 45, no. Pt 4 (2001): 300–307. 11489051 10.1046/j.1365-2788.2001.00322.x

[30] M. Sandberg , J. Kristensson , and A. Axmon , “Age‐Specific Diagnostic Panorama Among People With Intellectual Disabilities in Comparison With the General Population: A Longitudinal Register Study (IDcare),” Journal of Intellectual Disability Research 70 (2025): 295–315. 41408913 10.1111/jir.70072 PMC12872377

[31] H. Hasle , I. H. Clemmensen , and M. Mikkelsen , “Risks of Leukaemia and Solid Tumours in Individuals With Down's Syndrome,” Lancet 355, no. 9199 (2000): 165–169. 10675114 10.1016/S0140-6736(99)05264-2

[32] T. Sappok , M. L. Rosenbusch , R. Hering , et al., “Cancer Prevalence and Care Disparities Among Individuals With Intellectual Disabilities: A Cross‐Sectional Pan‐Cancer Analysis,” ESMO Real World Data and Digital Oncology 9 (2025): 100160. 41646220 10.1016/j.esmorw.2025.100160 PMC12836748

[33] Cancer Council , “Early Detection of Prostate Cancer,” (2022), https://www.cancer.org.au/cancer‐information/causes‐and‐prevention/early‐detection‐and‐screening/early‐detection‐of‐prostate‐cancer.

[34] A. J. Jiang , P. V. Rambhatla , and M. J. Eide , “Socioeconomic and Lifestyle Factors and Melanoma: A Systematic Review,” British Journal of Dermatology 172, no. 4 (2015): 885–915. 25354495 10.1111/bjd.13500

[35] C. H. Pui , L. L. Robison , and A. T. Look , “Acute Lymphoblastic Leukaemia,” Lancet 371, no. 9617 (2008): 1030–1043. 18358930 10.1016/S0140-6736(08)60457-2

[36] J. A. Ross , S. M. Davies , J. D. Potter , and L. L. Robison , “Epidemiology of Childhood Leukemia, With a Focus on Infants,” Epidemiologic Reviews 16, no. 2 (1994): 243–272. 7713179 10.1093/oxfordjournals.epirev.a036153

[37] A. Banda , J. Naaldenberg , A. Timen , A. van Eeghen , G. Leusink , and M. Cuypers , “Cancer Risks Related to Intellectual Disabilities: A Systematic Review,” Cancer Medicine 13, no. 9 (2024): e7210. 38686623 10.1002/cam4.7210 PMC11058689

[38] G. Young‐Southward , E. Rydzewska , C. Philo , and S. A. Cooper , “Physical and Mental Health of Young People With and Without Intellectual Disabilities: Cross‐Sectional Analysis of a Whole Country Population,” Journal of Intellectual Disability Research 61, no. 10 (2017): 984–993. 28895262 10.1111/jir.12422

[39] G. L. Krahn , L. Hammond , and A. Turner , “A Cascade of Disparities: Health and Health Care Access for People With Intellectual Disabilities,” Mental Retardation and Developmental Disabilities Research Reviews 12, no. 1 (2006): 70–82. 16435327 10.1002/mrdd.20098

[40] H. Ouellette‐Kuntz , N. Garcin , M. E. Lewis , P. Minnes , C. Martin , and J. J. Holden , “Addressing Health Disparities Through Promoting Equity for Individuals With Intellectual Disability,” Canadian Journal of Public Health 96, no. Suppl 2 (2005): S8–S22. 10.1007/BF03403699 PMC6976115 16078552

[41] S. Bhaumik , J. M. Watson , C. F. Thorp , F. Tyrer , and C. W. McGrother , “Body Mass Index in Adults With Intellectual Disability: Distribution, Associations and Service Implications: A Population‐Based Prevalence Study,” Journal of Intellectual Disability Research 52, no. Pt 4 (2008): 287–298. 18339091 10.1111/j.1365-2788.2007.01018.x

[42] G. M. de Kuijper and P. J. Hoekstra , “Physicians' Reasons Not to Discontinue Long‐Term Used Off‐Label Antipsychotic Drugs in People With Intellectual Disability,” Journal of Intellectual Disability Research 61, no. 10 (2017): 899–908. 28560761 10.1111/jir.12385

[43] C. A. Melville , S. Hamilton , C. R. Hankey , S. Miller , and S. Boyle , “The Prevalence and Determinants of Obesity in Adults With Intellectual Disabilities,” Obesity Reviews 8, no. 3 (2007): 223–230. 17444964 10.1111/j.1467-789X.2006.00296.x

[44] E. Emerson , C. Hatton , S. Baines , and J. Robertson , “The Physical Health of British Adults With Intellectual Disability: Cross Sectional Study,” International Journal for Equity in Health 15 (2016): 11. 26791808 10.1186/s12939-016-0296-x PMC4719222

[45] N. G. Lennox , C. E. Brolan , J. Dean , et al., “General Practitioners' Views on Perceived and Actual Gains, Benefits and Barriers Associated With the Implementation of an Australian Health Assessment for People With Intellectual Disability,” Journal of Intellectual Disability Research 57, no. 10 (2013): 913–922. 22774940 10.1111/j.1365-2788.2012.01586.x

[46] Centre for Health Record Linkage , “Quality Assurance,” (2026), https://www.cherel.org.au/quality‐assurance.

