# Supplementary material for: Cancer Incidence in People With Intellectual Disability and Down Syndrome in Australia: A Cohort Study
Source: Cancer Med. 2026 Apr 29;15(5):e71866. doi: 10.1002/cam4.71866 (PMC13129230; doi:10.1002/cam4.71866)
Supplement: Supplementary file 1 — Table S1: Summary of Population‐Based Studies on Cancer Incidence in People with Intellectual Disabilities. Table S2: Diagnostic codes for intellectual disability. Table S3: Cancer groupings. Table S4: Characteristics of people with cancer, by cancer type and intellectual disability status. Table S5: Cohort characteristics of people with intellectual disability with cancer by Down syndrome status. Table S6: Hazard ratios of any cancer in people with Down syndrome. Table S7: Cohort characteristics of individuals with and without missing information. Figure S1: Directed acyclic graph showing proposed causal relationships between predictor variables and the outcome diagnosis for the intellectual disability cohort. Figure made using DAGitty (http://www.dagitty.net/). Figure S2: Directed acyclic graph showing proposed causal relationships between predictor variables and the outcome diagnosis for the Down syndrome cohort. Figure made using DAGitty (http://www.dagitty.net/). [file CAM4-15-e71866-s001.docx]

| **Supplementary Table 1:** Summary of Population-Based Studies on Cancer Incidence in People with Intellectual Disabilities | | | | | | | |
| --- | --- | --- | --- | --- | --- | --- | --- |
| Study (Year) | Source of Cases | Source of Comparators | Source of Outcomes | Country | Covariates | Analytical Approach | Findings |
| Patja et al. (2001) | Population register of people with intellectual disability in Finland | General population | Finnish Cancer Registry | Finland | Age, sex | Standardized incidence ratios | Overall cancer incidence similar to general population; elevated for esophagus, stomach, gallbladder, kidney, CNS |
| Sullivan et al. (2004) | WA Disability Services Commission register | General WA population | WA Cancer Registry | Australia | Age, sex | Standardized incidence ratios | Overall cancer incidence lower than general population; higher for digestive system, brain, and testicular cancers |
| Sappok et al. (2024) | Medical records from specialist institutions | Not specified | Medical records | Germany | Not stated | Descriptive summary | Cancer occurred at younger ages; higher frequency of some cancer types, including digestive and hematological |
| Hosking et al. (2024) | Scottish Census 2011 (self/proxy report of ID) | General Scottish population | Cancer registry and death certificates | Scotland | Age, sex, deprivation | Age-standardized incidence and mortality ratios | Higher mortality in ID group despite similar or lower incidence; higher for colorectal, breast, and unknown primary cancers |
| Hoekstra et al. (2021) | Dutch national population register | General Dutch population | National mortality register | Netherlands | Age, sex | Mortality ratios | Cancer was 1.5x more likely cause of death in ID group; higher mortality from screening-related and digestive cancers |
| Sandberg et al. (2022) | Swedish national registries (birth cohort) | General Swedish population | National Cancer Registry | Sweden | Age, sex, birth year | Hazard ratios | Increased cancer risk in ID group (HR 1.6); elevated for GI, uterus, CNS, and hematological malignancies |
| Cuypers M et al. (2025) | National administrative databases from Statistics Netherlands (CBS). Adults (18+) identified via entitlements to long-term care or income benefits specifically for intellectual disability. | A 1:4 random sample of the Dutch adult population without indicators of intellectual disability, also sourced from Statistics Netherlands (CBS). | The Netherlands Cancer Registry (NCR), which records all new cancer diagnoses via notification from pathological, hospital, and laboratory databases. | The Netherlands | Age and sex. Subgroup analyses also considered living situation (residential care vs. independent living). | Population-based cohort study. Calculated person-years (PY) and crude incidence rates per 10,000 PY. Binomial logistic regression was used to calculate adjusted Odds Ratios (adj.OR) with 95% Confidence Intervals. | People with intellectual disability had a lower overall cancer incidence (adj.OR 0.79) but were diagnosed at younger ages and more advanced stages. They had lower odds of skin cancer (adj.OR 0.39) but higher odds of cancer of unknown primary (OR 1.60). Those living independently had higher risks for digestive and respiratory cancers than those in residential care. |

**Supplementary Table 2:** Diagnostic codes for intellectual disability

| Description | ICD-9 | ICD-10 |
| --- | --- | --- |
| Intellectual disability | 317, 318 (318.0, 318.1, 318.2), 319 | F70–F73, F78–F79 (all subcodes) |
| Other cerebral degenerations | 330.8 | - |
| Rett syndrome | - | F84.2 |
| Down syndrome | 758.0 | Q90 (all subcodes) |
| Edwards and Patau syndromes | 758.1, 758.2 | Q91 (all subcodes) |
| Other chromosomal abnormalities | 758, 758.31, 758.32, 758.33, 758.39 | Q93, Q95-Q99 (all subcodes) |
| Fetal alcohol syndrome | 760.71 | P04.3 |
| Other syndromes associated with intellectual disability | 759.81, 759.82, 759.83, 759.89 | Q86-Q87 (all subcodes), Q89.8 |

**Supplementary Table 3:** Cancer groupings

| Diagnosis | ICD-10 codes |
| --- | --- |
| All haematological cancers | C81 – C96 |
| All solid cancers | C00 – C76; C80 |
| Colorectal | C18 – C20 |
| Breast | C50 |
| Cervical | C53 |
| Melanoma | C43 |
| Prostate | C61 |
| Lung | C33 – C34 |
| Liver | C22 |

**Supplementary Table 4:** Characteristics of people with cancer, by cancer type and intellectual disability status.

|  | All cancers - Intellectual Disability | All cancers - Matched Comparator | All Solid - Intellectual Disability | All Solid - Matched comparator | All Haematological - Intellectual Disability | All Haematological - Matched Comparator |
| --- | --- | --- | --- | --- | --- | --- |
| N | 2,474 | 11,662 | 2,004 | 10,082 | 286 | 1,150 |
| Sex |  |  |  |  |  |  |
| Female | 1,155 (46.7%) | 4,918 (42.2%) | 965 (48.2%) | 4,321 (42.9%) | 110 (38.5%) | 427 (37.1%) |
| Male | 1,319 (53.3%) | 6,744 (57.8%) | 1,039 (51.8%) | 5,761 (57.1%) | 176 (61.5%) | 723 (62.9%) |
| Residential remoteness |  |  |  |  |  |  |
| Major cities | 1,571 (63.5%) | 7,041 (60.4%) | 1,277 (63.7%) | 6,061 (60.1%) | 180 (62.9%) | 718 (62.4%) |
| Inner regional | 506 (20.5%) | 2,642 (22.7%) | 410 (20.5%) | 2,299 (22.8%) | 60 (21.0%) | 252 (21.9%) |
| Outer regional | 152 (6.1%) | 1,014 (8.7%) | 124 (6.2%) | 872 (8.6%) | 13 (4.5%) | 100 (8.7%) |
| Remote & very remote | 17 (0.7%) | 81 (0.7%) | 15 (0.7%) | 76 (0.8%) | * | * |
| Missing | 228 (9.2%) | 884 (7.6%) | 178 (8.9%) | 774 (7.7%) | * | * |
| Index of Relative Socioeconomic Disadvantage (IRSD) | | |  |  |  |  |
| First quintile (most disadvantaged) | 502 (20.3%) | 2,234 (19.2%) | 419 (20.9%) | 1,896 (18.8%) | 46 (16.1%) | 236 (20.5%) |
| Second quintile | 473 (19.1%) | 2,371 (20.3%) | 385 (19.2%) | 2,045 (20.3%) | 52 (18.2%) | 245 (21.3%) |
| Third quintile | 584 (23.6%) | 2,686 (23.0%) | 477 (23.8%) | 2,333 (23.1%) | 71 (24.8%) | 266 (23.1%) |
| Fourth quintile | 376 (15.2%) | 1,967 (16.9%) | 297 (14.8%) | 1,700 (16.9%) | 52 (18.2%) | 189 (16.4%) |
| Fifth quintile (least disadvantaged) | 354 (14.3%) | 1,800 (15.4%) | 280 (14.0%) | 1,568 (15.6%) | 41 (14.3%) | 176 (15.3%) |
| Missing | 185 (7.5%) | 604 (5.2%) | 146 (7.3%) | 540 (5.4%) | 24 (8.4%) | 38 (3.3%) |
| Age at cancer diagnosis, years | |  |  |  |  |  |
| 0-4 | 144 (5.8%) | 242 (2.1%) | 73 (3.6%) | 117 (1.2%) | 51 (17.8%) | 82 (7.1%) |
| 5-14 | 97 (3.9%) | 260 (2.2%) | 56 (2.8%) | 119 (1.2%) | 31 (10.8%) | 101 (8.8%) |
| 15-18 | 53 (2.1%) | 142 (1.2%) | 31 (1.5%) | 96 (1.0%) | 13 (4.5%) | 42 (3.7%) |
| 19-24 | 51 (2.1%) | 213 (1.8%) | 32 (1.6%) | 157 (1.6%) | 11 (3.8%) | 49 (4.3%) |
| 25-44 | 430 (17.4%) | 1,378 (11.8%) | 351 (17.5%) | 1,202 (11.9%) | 49 (17.1%) | 148 (12.9%) |
| 45-64 | 1,093 (44.2%) | 5,321 (45.6%) | 945 (47.2%) | 4,806 (47.7%) | 87 (30.4%) | 385 (33.5%) |
| 65+ | 606 (24.5%) | 4,106 (35.2%) | 516 (25.7%) | 3,585 (35.6%) | 44 (15.4%) | 343 (29.8%) |
| Calendar year at cancer diagnosis | |  |  |  |  |  |
| 2002-2005 | 525 (21.2%) | 2,460 (21.1%) | 406 (20.3%) | 2,085 (20.7%) | 76 (26.6%) | 263 (22.9%) |
| 2006-2009 | 577 (23.3%) | 2,612 (22.4%) | 484 (24.2%) | 2,261 (22.4%) | 55 (19.2%) | 258 (22.4%) |
| 2010-2013 | 686 (27.7%) | 3,151 (27.0%) | 549 (27.4%) | 2,741 (27.2%) | 76 (26.6%) | 305 (26.5%) |
| 2014-2017 | 686 (27.7%) | 3,439 (29.5%) | 565 (28.2%) | 2,995 (29.7%) | 79 (27.6%) | 324 (28.2%) |

**Supplementary Table 5:** Cohort characteristics of people with intellectual disability with cancer by Down syndrome status

|  | Down syndrome  (n=145) | Intellectual disability without Down syndrome  (n=2,329) |
| --- | --- | --- |
| Sex |  |  |
| Female | 67 (46.2%) | 1,088 (46.7%) |
| Male | 78 (53.8%) | 1,241 (53.3%) |
| Residential remoteness |  |  |
| Major cities | 91 (62.8%) | 1,480 (63.5%) |
| Inner regional | 26 (17.9%) | 480 (20.6%) |
| Outer regional | * | 145 (6.2%) |
| Remote & very remote | * | 16 (0.7%) |
| Missing | 20 (13.8%) | 208 (8.9%) |
| Index of Relative Socioeconomic Disadvantage (IRSD) | |  |
| First quintile (most disadvantaged) | 25 (17.2%) | 477 (20.5%) |
| Second quintile | 25 (17.2%) | 448 (19.2%) |
| Third quintile | 24 (16.6%) | 560 (24.0%) |
| Fourth quintile | 19 (13.1%) | 357 (15.3%) |
| Fifth quintile (least disadvantaged) | 35 (24.1%) | 319 (13.7%) |
| Missing | 17 (11.7%) | 168 (7.2%) |
| Age at diagnosis, years |  |  |
| 0-4 | 35 (24.1%) | 109 (4.7%) |
| 5-14 | 10 (6.9%) | 87 (3.7%) |
| 15-18 | * | * |
| 19-24 | * | * |
| 25-44 | 37 (25.5%) | 393 (16.9%) |
| 45-64 | 44 (30.3%) | 1,049 (45.0%) |
| 65+ | 11 (7.6%) | 595 (25.5%) |
| Calendar period at diagnosis |  |  |
| 2002-2005 | 48 (33.1%) | 477 (20.5%) |
| 2006-2009 | 22 (15.2%) | 555 (23.8%) |
| 2010-2013 | 38 (26.2%) | 648 (27.8%) |
| 2014-2017 | 37 (25.5%) | 649 (27.9%) |

Note: Missing category excluded from the statistical analysis, * excluded as cell count < 6

| **Supplementary Table 6:** Hazard ratios of any cancer in people with Down syndrome | |
| --- | --- |
|  | **SHR (95% CI)** |
| **All ages** | |
| Down syndrome vs. other intellectual disability | 1.03 (0.87-1.21) |
| **0-14 years** | |
| Down syndrome vs. other intellectual disability | 7.94 (5.72-11.04) |
| **15-49 years** | |
| Down syndrome vs. other intellectual disability | 1.08 (0.82-1.43) |
| **50+** | |
| Down syndrome vs. other intellectual disability | 0.46 (0.46-0.82) |

Note: All models were adjusted for sex, Indigenous status, remoteness and area-level socio-economic status.

SHR - sub hazard ratio

CI - confidence intervals

Age is a timescale variable for all analyses.

**Supplementary Table 7:** Cohort characteristics of individuals with and without missing information

|  | **Intellectual disability (included)** | **Intellectual disability (excluded)** | **Matched comparator (included)** | **Matched comparator (excluded)** |
| --- | --- | --- | --- | --- |
| **Total (%)** | 99,897 (18,0%) | 6,157 (18.9%) | 455,434 (82.0%) | 26,342 (81.1%) |
| **Age at study start, years** | | | | |
| 0-14 | 70,825 (70.9%) | 4,616 (75.0%) | 317,391 (69.7%) | 18,472 (70.1%) |
| 15-18 | 3,450 (3.5%) | 270 (4.4%) | 17,812 (3.9%) | 1,544 (5.9%) |
| 19-24 | 4,170 (4.2%) | 289 (4.7%) | 20,845 (4.6%) | 1,703 (6.5%) |
| 25-44 | 12,903 (12.9%) | 700 (11.4%) | 58,189 (12.8%) | 3,226 (12.2%) |
| 45-64 | 7,087 (7.1%) | 248 (4.0%) | 33,884 (7.4%) | 1,253 (4.8%) |
| 65+ | 1,462 (1.5%) | 34 (0.6%) | 7,313 (1.6%) | 144 (0.5%) |
| **Sex** | | | | |
| Female | 33,730 (33.8%) | 2,075 (33.7%) | 159,129 (34.9%) | 9,581 (36.4%) |
| Male | 66,167 (66.2%) | 4,082 (66.3%) | 296,305 (65.1%) | 16,761 (63.6%) |
| **Area-level Index of Relative Socioeconomic Disadvantage (IRSD)** | | | | |
| First quintile  (most disadvantaged) | 24,629 (24.7%) | 1,548 (25.1%) | 102,743 (22.6%) | 4,958 (18.8%) |
| Second quintile | 22,057 (22.1%) | 1,475 (24.0%) | 99,407 (21.8%) | 5,803 (22.0%) |
| Third quintile | 21,139 (21.2%) | 1,189 (19.3%) | 97,831 (21.5%) | 5,853 (22.2%) |
| Fourth quintile | 15,358 (15.4%) | 1,212 (19.7%) | 78,585 (17.3%) | 5,859 (22.2%) |
| Fifth quintile  (least disadvantaged) | 11,645 (11.7%) | 733 (11.9%) | 62,115 (13.6%) | 3,869 (14.7%) |

Note: Remoteness could not be compared as this was the primary source of missingness. Down syndrome status not presented due to small numbers.

| **Supplementary Figure 1:** Directed acyclic graph showing proposed causal relationships between predictor variables and the outcome diagnosis for the intellectual disability cohort. Figure made using DAGitty (<http://www.dagitty.net/>)  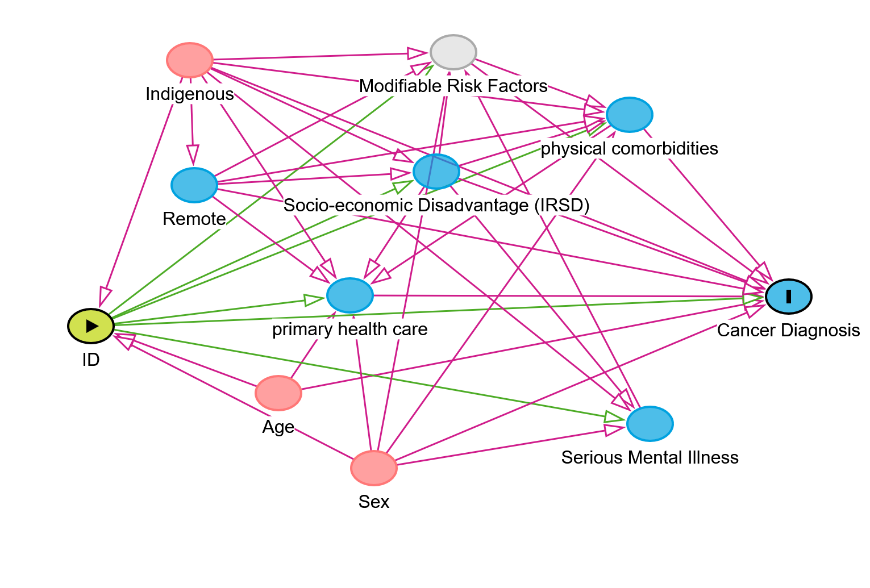 |
| --- |
|  |

Note: Green node = exposure (intellectual disability); blue node with "I" = outcome (cancer diagnosis); red nodes = adjusted confounders (age, sex, socioeconomic disadvantage); blue nodes = not adjusted (mediators or consequences of exposure); grey node = unmeasured (modifiable risk factors).

| **Supplementary Figure 2:** Directed acyclic graph showing proposed causal relationships between predictor variables and the outcome diagnosis for the Down syndrome cohort. Figure made using DAGitty (<http://www.dagitty.net/>) |
| --- |
| 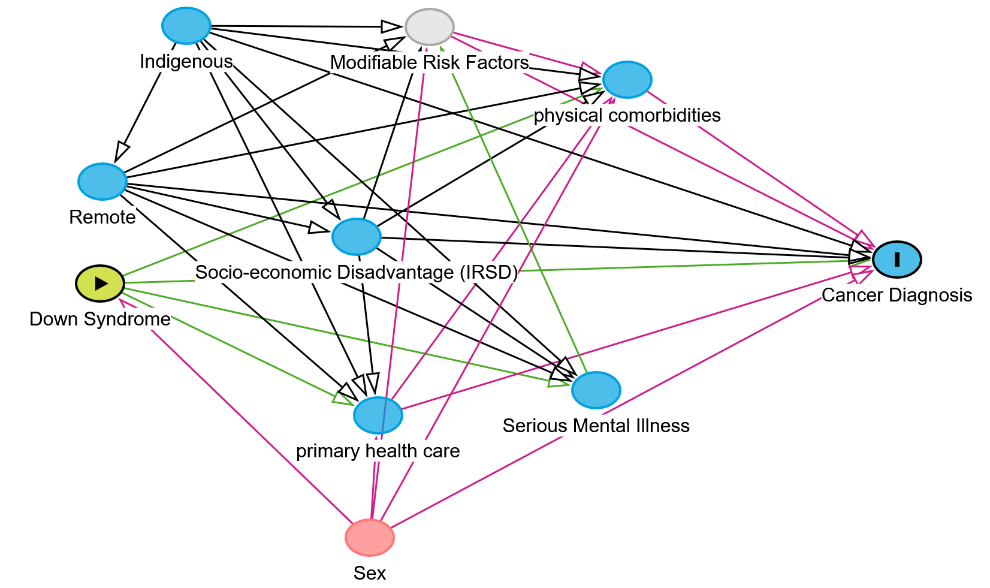 |

Note: Green node = exposure (Down syndrome); blue node with "I" = outcome (cancer diagnosis); red node = adjusted confounder (sex); blue nodes = not adjusted (Indigenous status, remoteness, socioeconomic disadvantage, primary health care, physical comorbidities, serious mental illness); grey node = unmeasured (modifiable risk factors).
